# An Unusual Presentation of Hyponatremia in a Premature Infant With Failure to Thrive

**DOI:** 10.7759/cureus.79911

**Published:** 2025-03-02

**Authors:** Aaska Patel, Amrita A Gujar, Louisdon Pierre

**Affiliations:** 1 Pediatrics, The Brooklyn Hospital Center, Brooklyn, USA

**Keywords:** electrolytes, failure to thrive, hyponatremia in icu, pediatrics, premature

## Abstract

Hyponatremia in neonates is a rare but not uncommon finding, especially among preterm neonates, and can be life-threatening, requiring careful diagnostic evaluation and management. We describe a two-month-old infant born at 33 weeks of gestation presenting with severe hyponatremia and failure to thrive (FTT) to highlight the diagnostic and management approach and challenges in such cases.

A two-month-old male child presented with abdominal distension and poor weight gain. Admission weight was 2.72 kgs (<3rd percentile). Lab results revealed a serum sodium of 108 mEq/L and potassium of 2.6 mEq/L. X-ray abdomen and ultrasounds were unremarkable for biliary or gastrointestinal obstruction. Electrolyte abnormalities were successfully corrected with potassium chloride and normal saline. This case demonstrates the importance of assessing feeding adequacy, renal salt wasting, and potential endocrine/metabolic causes in infants with FTT and electrolyte disturbances. Timely recognition and targeted therapy for neonatal and infantile hyponatremia are crucial to prevent complications.

## Introduction

Neonatal and infantile hyponatremia is a rare but not uncommon finding, especially among preterm neonates, and can be life-threatening, requiring careful diagnostic evaluation and management [1]. Failure to thrive (FTT) in neonates and infants is a concerning presentation that may result from a wide spectrum of conditions, from inadequate caloric intake to complex metabolic or genetic disorders. Early recognition and intervention are crucial to prevent long-term growth and developmental sequelae. Severe hyponatremia in this age group is rare and often associated with significant underlying pathology. The disturbance in this population may result from improper feeding practices, renal losses, or endocrine dysfunction, such as congenital adrenal hyperplasia or pseudohypoaldosteronism [2]. The concurrent findings of abdominal distension and growth failure in this patient further expand the differential diagnosis to include gastrointestinal and metabolic causes. The combination of persistent electrolyte abnormalities, poor growth, and abdominal distension suggests a multifactorial etiology involving nutritional, renal, or metabolic components [3]. While improper feeding practices may explain the growth failure, the severity of the hyponatremia raises suspicion for an underlying renal or endocrine disorder, necessitating further investigation. This case highlights the diagnostic challenges posed by non-specific presentations of FTT and hyponatremia in a two-month-old infant. It underscores the importance of considering rare and complex conditions, including genetic and metabolic disorders, while systematically addressing common causes like feeding inadequacy.

## Case presentation

A two-month-old male child, born at 33 weeks of gestation via normal spontaneous vaginal delivery with a 35-day neonatal intensive care unit (NICU) stay for prematurity with a birth weight of 2251 g (73rd percentile per Fenton growth chart), presented for a two-month well-child clinic visit with FTT (weight at 1.82 percentile per Fenton growth chart, 2722 g) and abdominal distension. Feeding history by the mother reported administration of 3 oz (89 mL) of 22 kcal formula and breast milk every 2-3 hours, totaling 12-14 oz (355-414 mL)/day. The infant produced 6-8 wet diapers daily. She reported no fevers, respiratory symptoms, diarrhea, vomiting, or recent travel. No known family history of any congenital anomalies or neonatal deaths.

During the NICU course, the infant required positive pressure ventilation and continuous positive airway pressure at birth for apnea, failed a room air trial on day two, received a high-flow nasal cannula, and weaned to room air on day three. Sepsis was ruled out after three days of antibiotics. A heart murmur was noted on day 25; an echocardiogram showed physiologic peripheral pulmonary stenosis, which was not concerning. Phototherapy was given from day 2 to day 8 for a bilirubin peak of 11.6 mg/dl. Persistent abdominal distension prompted imaging and consultations, but no surgical intervention was needed. The infant fed well on expressed breast milk fortified to 22 kcal per oz by mouth every three hours. A neurological examination was normal, and head ultrasounds were within normal limits. The infant was discharged in stable condition with outpatient follow-up for gastrointestinal care.

The initial vital signs were unremarkable with a temperature of 97.5°F (36.4°C), heart rate of 135 beats per minute, blood pressure of 85/59 mmHg, respiratory rate of 35 breaths per minute, and SpO_2_ 100%. Physical exam was remarkable for abdominal distension, with no tenderness, organomegaly, or palpable masses. Initial labs revealed severe hyponatremia (Na 107 mEq/L), hypokalemia (K 2.8 mEq/L), and hypochloremia (Cl 61 mEq/L), which prompted admission to the pediatric intensive care unit. The management focused on the gradual correction of electrolyte abnormalities with sodium chloride solution via nasogastric (NG) tube and intravenous potassium chloride supplementation. Sodium levels improved incrementally to 136 mEq/L, and the potassium increased to 4.3 mEq/L (Table 1).

**Table 1:** Electrolytes level trend L: low; LL: extremely low

	Sodium	Potassium	Chloride	Potassium, random urine	Sodium, random urine	Chloride, random urine
Latest reference range	136-145 mmol/L	3.5-5.1 mmol/L	98-107 mmol/L	25.0-125.0 mmol/L	40-220 mmol/L	110-250 mmol/L
July 24, 2024	107 (LL)	3.2 (L)	61 (L)			
	107 (LL)	2.9 (L)	60 (L)			
July 25, 2024	108 (LL)	2.6 (LL)	61 (L)	8.9 (L)	<20 (L)	<20 (L)
	115 (LL)	3.2 (L)	71 (L)	16.5 (L)	<20 (L)	<20 (L)
	120 (L)	3.2 (L)	79 (L)			
	122 (L)	2.2 (LL)	81 (L)			
July 26, 2024	126 (L)	3.8	86 (L)			
July 27, 2024	128 (L)	4.1	90 (L)			
	132 (L)	4.7	94 (L)			
July 28, 2024	136	3.8	95 (L)			
	134 (L)	4.2	95 (L)			
July 29, 2024	138	4	97 (L)			
July 30, 2024	136	4.3	97 (L)			

Nephrology, gastroenterology, and endocrinology consultations did not reveal a cause, with all relevant investigations including serum aldosterone, renin, and cortisol levels, all within normal limits. The patient showed gradual weight gain during the course of hospitalization and was discharged home on sodium chloride supplementation.

The patient was admitted four times in six months for persistent hyponatremia. A sweat chloride test for cystic fibrosis was negative. Outpatient genetic evaluation was recommended as it is unavailable at our institution, and the etiology of hyponatremia remained unclear.

Despite a comprehensive workup, including GI and renal evaluations, the cause of the patient’s recurrent severe hyponatremia and FTT remains unknown. He requires sodium supplementation and ongoing evaluations with GI and genetic specialists.

## Discussion

This case highlights the importance and challenges of evaluation and management of severe hyponatremia in early infancy. When the usual workup is negative, it is important to consider genetic causes like congenital sodium diarrhea (CSD), congenital chloride diarrhea (CCD), or epithelial sodium channel (ENaC) disorders in infants presenting with salt-wasting and FTT. We discuss a two-month-old ex-33-week premature infant presenting with severe hyponatremia (108 mEq/L), hypokalemia (2.6 mEq/L), and FTT. The concurrent abdominal distension and electrolyte disturbances suggested a gastrointestinal origin of salt loss, warranting consideration of rare genetic disorders such as CCD and CSD.

CCD is caused by mutations in the SLC26A3 gene, leading to defective chloride-bicarbonate exchange in the intestines [1]. Clinical features include chronic diarrhea, metabolic alkalosis, hypokalemia, and significant fecal chloride losses. In this patient, the severe electrolyte imbalances (Cl 61 mEq/L) and history of abdominal distension align with CCD. Although diarrhea was not reported, its absence does not rule out CCD, as stool frequency may be misreported in neonates [2,3]. Additionally, SLC26A3 mutations exhibit phenotypic variability, making diagnosis challenging [4]. Management typically includes electrolyte replacement, chloride-rich formulas, and, in some cases, bicarbonate therapy [5].

Another condition to consider is CSD, which is caused by mutations in the SPINT2 gene, leading to sodium malabsorption in the gut [6]. Clinical features include chronic watery diarrhea, profound hyponatremia, and FTT. The profound hyponatremia (108 mEq/L) and abdominal findings are consistent with CSD, though the absence of reported diarrhea makes this diagnosis less likely [7]. Genetic testing could help confirm or exclude SPINT2 mutations. Studies have highlighted the variability in CSD presentations, with some cases manifesting with minimal diarrhea, further complicating the diagnostic approach [8].

Furthermore, mutations in the ENaC can lead to pseudohypoaldosteronism type 1 (PHA1), which can fit this patient’s presentation [9]. ENaC mutations result in impaired sodium reabsorption in epithelial tissues, leading to salt-wasting syndromes that manifest with hyponatremia (108 mEq/L), hyperkalemia (2.6 mEq/L), and FTT. Loss-of-function mutations impair sodium reabsorption, leading to profound salt loss through urine, sweat, and stool. This systemic dysfunction, characteristic of autosomal recessive pseudohypoaldosteronism type 1 (PHA1B), typically presents in infancy with severe hyponatremia, hyperkalemia, and FTT [10]. However, hypokalemia in this case may reflect compensatory mechanisms from chronic salt loss or dietary insufficiency. Abdominal distension could be secondary to fluid retention in the intestinal lumen due to impaired sodium transport. The patient's prematurity (33 weeks) may have further exacerbated sodium losses, as immature kidneys have reduced capacity to compensate for electrolyte imbalances. Additionally, the feeding history raised concerns about potential compounding nutritional deficiencies, but these alone are unlikely to account for the severe electrolyte disturbances. Variability in ENaC mutations can lead to a spectrum of clinical severity, necessitating genetic testing for confirmation [11].

An additional condition to consider is Pseudo-Bartter syndrome, which is often associated with significant electrolyte losses and may mimic other salt-wasting disorders. This syndrome, which has been reported in cases of CCD, presents with metabolic alkalosis, dehydration, and FTT [12]. It is commonly observed in conditions like cystic fibrosis but can also be seen in severe gastrointestinal and renal salt-wasting disorders [13]. Given this patient's clinical presentation, it is essential to differentiate among these potential diagnoses to guide management. Table 2 provides a comparative analysis of these differential diagnoses [14].

**Table 2:** Comparison of differential diagnoses for neonatal salt-wasting disorders Reference: [14] ENaC: epithelial sodium channel

Feature	Congenital chloride diarrhea (CCD)	Congenital sodium diarrhea (CSD)	Pseudohypoaldosteronism type 1 (PHA1)	Pseudo-Bartter syndrome (PBS)
Genetic cause	SLC26A3 mutation	SPINT2 mutation	SCNN1A, SCNN1B, SCNN1G mutations	Secondary to cystic fibrosis, diarrhea, diuretics
Inheritance pattern	Autosomal recessive	Autosomal recessive	Autosomal recessive/dominant	Acquired/secondary
Primary defect	Impaired Cl⁻/HCO₃⁻ exchange in intestines	Sodium malabsorption in intestines	Defective ENaC function	Secondary to chronic fluid loss
Serum sodium (Na⁺)	Normal to low	Severely low (<110 mEq/L)	Severely low (<110 mEq/L)	Normal to low
Serum potassium (K⁺)	Low	Low	High	Low
Serum chloride (Cl⁻)	Low	Normal to low	Normal	Low
Serum bicarbonate (HCO₃⁻)	High (metabolic alkalosis)	Variable	Low to normal (metabolic acidosis)	High (metabolic alkalosis)
Fecal electrolytes	High Cl⁻ loss	High Na⁺ loss	Normal	Normal
Urinary sodium (UNa⁺)	Low	Low	High	Low
Urinary potassium (UK⁺)	Low	Low	High	Low
Diarrhea presence	Profuse, watery diarrhea	Watery diarrhea	Absent	Absent
Abdominal distension	Present	Present	Absent	Absent
Response to aldosterone	Normal	Normal	Absent (resistance)	Normal
Treatment	Electrolyte supplementation, chloride-rich formula	Sodium supplementation	Sodium supplementation, fludrocortisone	Treat the underlying cause, fluid/electrolyte replacement

## Conclusions

This case highlights the importance of considering rare genetic causes, such as ENaC mutations associated with systemic PHA1, in neonates and infants presenting with severe hyponatremia, electrolyte imbalances, and FTT. Early recognition and targeted management, including sodium supplementation and long-term monitoring, are critical to improving outcomes. This case underscores the need for a systematic approach to diagnostic evaluation in complex presentations to guide appropriate therapy and provide families with valuable genetic insights.

## References

[1] Höglund P, Auranen M, Socha J (1998). Genetic background of congenital chloride diarrhea in high-incidence populations: Finland, Poland, and Saudi Arabia and Kuwait. Am J Hum Genet.

[2] Wedenoja S, Pekansaari E, Höglund P, Mäkelä S, Holmberg C, Kere J (2011). Update on SLC26A3 mutations in congenital chloride diarrhea. Hum Mutat.

[3] Alves C, Diederichsen S, Lund C (2020). Congenital chloride diarrhea: case report and review of the literature. Eur J Pediatr.

[4] Kere J, Lohi H, Höglund P (2004). Molecular genetics of congenital chloride diarrhea. Ann Med.

[5] Canani RB, Castaldo G, Bacchetta R (2001). Congenital chloride diarrhea: clinical findings and management in infancy. Clin Pediatr.

[6] Heinz-Erian P, Müller T, Krabichler B (2009). Mutations in SPINT2 cause a syndromic form of congenital sodium diarrhea. Am J Hum Genet.

[7] Müller T, Wijmenga C, Phillips AD (2000). Congenital sodium diarrhea is an autosomal recessive disorder of sodium/proton exchange but unrelated to known candidate genes. Gastroenterology.

[8] Ozen H, Dalgic B (2011). Congenital sodium diarrhea: a rare cause of neonatal diarrhea. Eur J Pediatr.

[9] Edelheit O, Hanukoglu I, Gizewska M (2005). Novel mutations in epithelial sodium channel (ENaC) subunit genes and phenotypic expression of multisystem pseudohypoaldosteronism. Clin Endocrinol (Oxf).

[10] Hanukoglu A, Edelheit O, Shriki Y (2008). Pseudohypoaldosteronism type 1: a multi-system disorder. Pediatr Nephrol.

[11] Saxena A, Amin HJ, Schlenzig JS (2012). Spectrum of epithelial sodium channel (ENaC) mutations in pseudohypoaldosteronism type 1. J Pediatr Endocrinol Metab.

[12] Stenström G, Vahlquist A (1992). Pseudo-Bartter syndrome in cystic fibrosis: pathogenetic role of chloride loss. J Pediatr.

[13] Kennedy JD, Dinwiddie R, Daman-Willems C, Dillon MJ, Matthew DJ (1990). Pseudo-Bartter's syndrome in cystic fibrosis. Arch Dis Child.

[14] Bizzarri C, Olivini N, Pedicelli S, Marini R, Giannone G, Cambiaso P, Cappa M (2016). Congenital primary adrenal insufficiency and selective aldosterone defects presenting as salt-wasting in infancy: a single center 10-year experience. Ital J Pediatr.

